# Quadriceps Muscle Fatigue Reduces Extension and Flexion Power During Maximal Cycling

**DOI:** 10.3389/fspor.2021.797288

**Published:** 2022-01-07

**Authors:** Steven J. O'Bryan, Janet L. Taylor, Jessica M. D'Amico, David M. Rouffet

**Affiliations:** ^1^Institute for Health and Sport (IHeS), Victoria University, Melbourne, VIC, Australia; ^2^Neuroscience Research Australia, Randwick, NSW, Australia; ^3^School of Medical and Health Sciences, Edith Cowan University, Perth, WA, Australia; ^4^Department of Neurological Surgery, University of Louisville, Louisville, KY, United States; ^5^Kentucky Spinal Cord Injury Research Center, University of Louisville, Louisville, KY, United States; ^6^Department of Health and Sport Sciences, University of Louisville, Louisville, KY, United States

**Keywords:** sprint, locomotion, electromyography, *rectus femoris*, *vastii* muscles, motor control, central fatigue, peripheral fatigue

## Abstract

**Purpose:** To investigate how quadriceps muscle fatigue affects power production over the extension and flexion phases and muscle activation during maximal cycling.

**Methods:** Ten participants performed 10-s maximal cycling efforts without fatigue and after 120 bilateral maximal concentric contractions of the quadriceps muscles. Extension power, flexion power and electromyographic (EMG) activity were compared between maximal cycling trials. We also investigated the associations between changes in quadriceps force during isometric maximal voluntary contractions (IMVC) and power output (flexion and extension) during maximal cycling, in addition to inter-individual variability in muscle activation and pedal force profiles.

**Results:** Quadriceps IMVC (−52 ± 21%, *P* = 0.002), voluntary activation (−24 ± 14%, *P* < 0.001) and resting twitch amplitude (−45 ± 19%, *P* = 0.002) were reduced following the fatiguing task, whereas vastus lateralis (*P* = 0.58) and vastus medialis (*P* = 0.15) M-wave amplitudes were unchanged. The reductions in extension power (−15 ± 8%, *P* < 0.001) and flexion power (−24 ± 18%, *P* < 0.001) recorded during maximal cycling with fatigue of the quadriceps were dissociated from the decreases in quadriceps IMVC. Peak EMG decreased across all muscles while inter-individual variability in pedal force and EMG profiles increased during maximal cycling with quadriceps fatigue.

**Conclusion:** Quadriceps fatigue induced by voluntary contractions led to reduced activation of all lower limb muscles, increased inter-individual variability and decreased power production during maximal cycling. Interestingly, power production was further reduced over the flexion phase (24%) than the extension phase (15%), likely due to larger levels of peripheral fatigue developed in RF muscle and/or a higher contribution of the quadriceps muscle to flexion power production compared to extension power during maximal cycling.

## Introduction

The maximal force-generating capacity of the quadriceps muscles is an important determinant of performance in numerous locomotor tasks of maximal intensity such as sprint running or maximal cycling. More specifically, individuals displaying larger force-generating capacity of their quadriceps muscles can reach higher speeds during sprint running (Lehance et al., 2009) and produce larger levels of power during maximal cycling (Driss et al., 2002; Kordi et al., 2017). During maximal cycling, the maximal force-generating capacity of the quadriceps is a strong determinant of power production over the extension phase with mono-articular *vastii* (VAS) muscles contributing ~35% of the total mechanical energy (Raasch et al., 1997) through knee extension power (McDaniel et al., 2014). The bi-articular *rectus femoris* (RF) muscle contributes to both knee extension power and hip flexion power, and it generates considerably more mechanical energy during the flexion phase compared to the extension phase; i.e. ~50 vs. ~5% (van Ingen Schenau, 1989; Raasch et al., 1997; McDaniel et al., 2014). Therefore, any reductions in force production by the quadriceps muscle should theoretically reduce power production over both the extension and flexion phases of the pedaling movement.

During prolonged cycling exercise of maximal or near-maximal intensity, fatigue develops for the quadriceps muscle as evidenced by reductions in isometric maximal voluntary contraction (IMVC) force. Fatigue reduced VAS and RF EMG activity and decreased knee extension power, which ultimately reduces crank power production during maximal cycling (Martin and Brown, 2009; O'Bryan et al., 2014, 2017). However, fatigue of the other lower limb muscles is also likely to develop during intense cycling as most lower limb muscles are activated at maximal or near-maximal levels (Rouffet and Hautier, 2008; Dorel et al., 2012; O'Bryan et al., 2014; Rudsits et al., 2018). In order to determine the effect of quadriceps fatigue on power production during maximal cycling, fatigue of the quadriceps muscle was induced using electrostimulation to create a ~30% reduction in unilateral IMVC of the quadriceps prior to a maximal cycling effort (Brochner Nielsen et al., 2018). Using this fatiguing protocol, impairment of contractile properties of VAS, and RF muscles likely caused most of the reductions in the force produced by the quadriceps muscles. Peripheral fatigue of the quadriceps was accompanied by reduced activation of the extensor muscles, which led to a ~9% decrease in extension phase power for the fatigued leg during maximal cycling. Unexpectedly, the authors also reported a ~20% increase in flexion phase power for the fatigued leg during maximal cycling when peripheral fatigue and a 17% decreased activation (Brochner Nielsen et al., 2018) should have theoretically reduced RF contribution to the flexion phase. Because RF muscle contributes ~50% of mechanical energy generated over the flexion phase (van Ingen Schenau, 1989; Raasch et al., 1997; McDaniel et al., 2014), a reduced contribution of RF should theoretically have decreased power production over the flexion phase. Moreover, despite the use of a high stimulation frequency, i.e., 70 Hz (Grosprêtre et al., 2017), the fatiguing protocol used by Brochner Nielsen et al. (2018) did not cause central fatigue that typically accompanies voluntary contractions (Babault et al., 2006; Morel et al., 2015). Thus, quadriceps fatigue induced in more ecological conditions may affect muscle activation of power production differently. Voluntary fatiguing contractions of the quadriceps are likely to alter the cortico-motor responses (Takahashi et al., 2011; Sidhu et al., 2014) and force production by non-exercised lower limbs muscles (Halperin et al., 2014). Interestingly, previous studies that used voluntary contractions to induce quadriceps fatigue reported a decreased activation of the hamstring muscles when tested during knee flexion isometric contractions (Amann et al., 2011; Kennedy et al., 2015). Theoretically, this phenomenon could also reduce power production over the flexion phase during maximal cycling. Additionally, fatigue of the quadriceps muscles is also likely to cause an increased variability of the motor command to the lower limb muscles, which could further reduce power production during maximal cycling (Rudsits et al., 2018). Therefore, the impact of quadriceps fatigue caused by voluntary contraction on muscle activation and power production during maximal cycling remains to be tested.

The first aim of this study was to determine the effect of a series of bilateral and voluntary knee extensions on quadriceps fatigue as well as activation of the lower-limb muscles and power production during a maximal cycling effort. We hypothesized that voluntary contractions would induce fatigue of central and peripheral origins, resulting into a decreased activation of non-exercised muscles and reductions in power production over both the extension and the flexion phases of the pedal cycle during maximal cycling with fatigued quadriceps. The second aim was to investigate the association between quadriceps IMVC and power production over the extension and flexion phases during maximal cycling with fatigued quadriceps. We hypothesized that IMVC of the quadriceps would be a strong determinant of power production during both phases of the pedal cycle in absence of quadriceps fatigue, whereas these associations would be weakened due to a reduced activation of other lower limb muscles and an increased variability in the motor control pattern between individuals during maximal cycling in the presence of quadriceps fatigue of central and peripheral origins.

## Methods

### Participants and Ethics

For participation in this study, six males and four females (*n* = 10) accustomed to regular high-intensity exercise were recruited (age: 26 ± 4 years; height: 179 ± 6 cm; mass: 74 ± 11 kg; mean ± SD). Sample size calculations using G^*^Power software (version 3.0.10) determined a required sample size of *n* = 8 based on an alpha level of 0.05, a power level of 0.95, and the assumption that the fatiguing task would lead to large reductions in maximal force of the quadriceps during knee extension IMVC and maximal cycling power (effect size *f* > 0.6) (Babault et al., 2006; O'Bryan et al., 2017). Participants were free from any existing cardiovascular, musculoskeletal or neuromuscular disorders. Written informed consent was obtained from each participant, and the study was approved by Victoria University's Human Research Ethics Committee (HRE14-82) in accordance with the standards set by the Declaration of Helsinki apart from registration of the study in a database.

### Experimental Protocol

**Figure 1:**
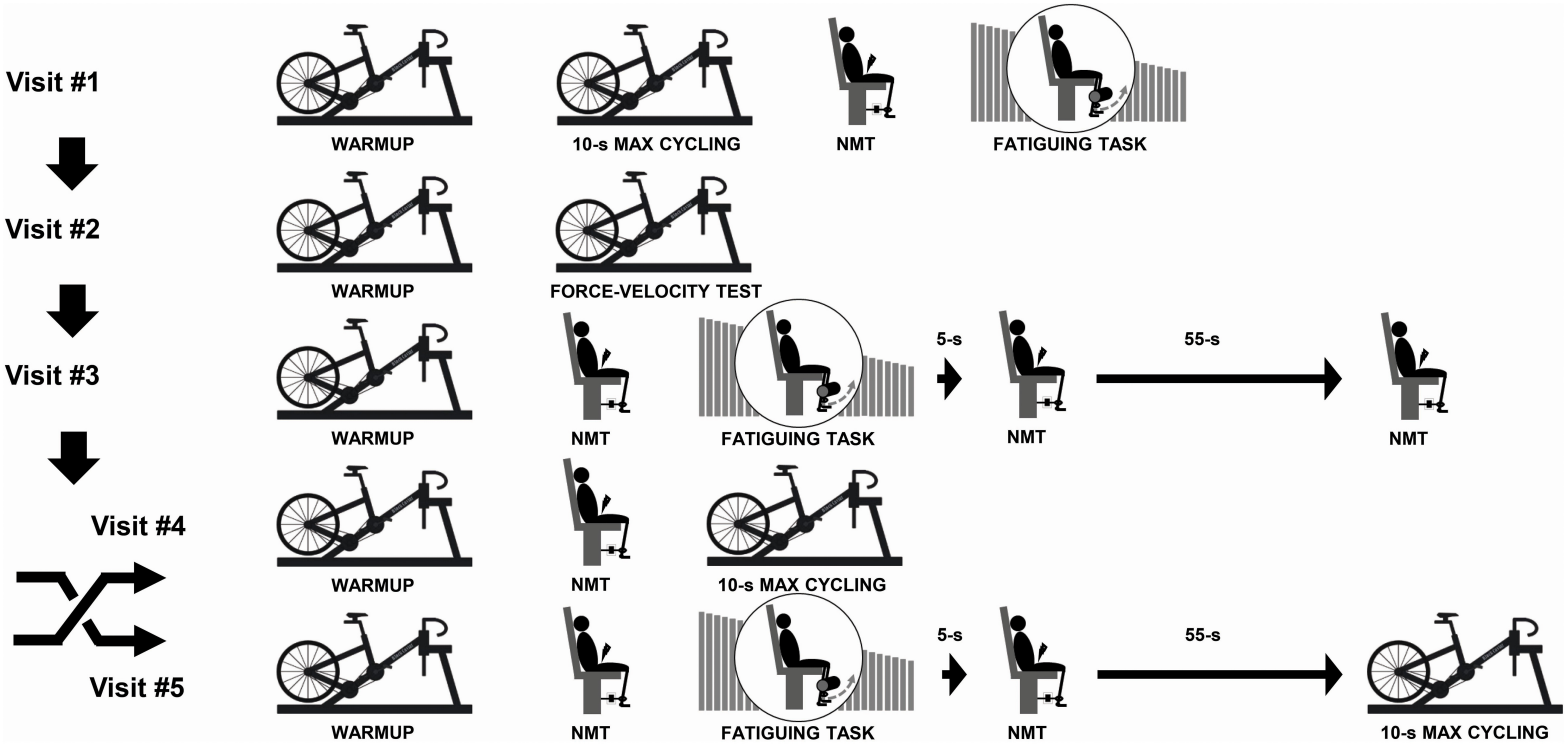
Illustration of the experimental protocol. During visit #1, participants were familiarized with the 10-s maximal cycling trial, the neuromuscular testing (NMT) procedures and the fatiguing task. During visit #2, we determined the power-cadence relationships for both legs and both phases (extension/flexion) in all participants while determining the gear ratio to be used for visits #4 and #5. During visit #3, we assessed fatigue of the knee extensors at 5 and 60 s post-fatiguing task to coincide with the time that participants would begin the 10-s maximal cycling trial during visit #4 or visit #5. The order of visits #4 and #5 was randomized and counterbalanced.

Participants visited the laboratory on five separate occasions (Figure 1). All visits were scheduled at the same time of day (±2 h) with at least 72 h between visits. Participants were instructed to avoid any strenuous physical activity during the 24 h preceding each visit. All visits were initiated by a 15-min standardized warm up on a stationary cycle ergometer (alternating between 4 and 5 min submaximal cycling and 4–6 s maximal efforts) followed by 5 min of passive rest.

During the first session, participants were familiarized with maximal cycling trials and neuromuscular testing (NMT) procedures to ensure test-retest reliability. During the second session, participants completed a Force-Velocity (F-V) cycling test to determine the power-cadence relationships of the two legs over the extension and flexion phases (Rudsits et al., 2018). Briefly, participants performed four to six 5–6 s maximal efforts in a randomized order over the following cadence ranges:(i) 23 ± 3 to 49 ± 13 rpm; (ii) 52 ± 13 to 109 ± 19 rpm; (iii) 67 ± 14 to 132 ± 18 rpm; (iv) 98 ± 28 to 164 ± 15 rpm; and (v) 123 ± 21 to 190 ± 17 rpm. During the third visit, participants completed a fatiguing task of 120 maximal bilateral voluntary contractions of their quadriceps during concentric knee extension exercise. NMT procedures were performed before, 5 and 60 s after the last contraction to determine the time course of recovery from the fatiguing task, and to verify that fatigue would still be present after the participants transferred to the stationary cycle ergometer to start the maximal cycling trial. During the fourth and fifth visits, participants completed a standardized warm-up procedure after which they either performed a brief maximal cycling trial, or the fatiguing task immediately before a brief maximal cycling trial. The order of the last two visits was randomized across participants. The maximal cycling trials were performed in iso-inertial mode with the same gear ratio used for each participant across the visits.

### Materials and Procedures

#### Maximal Cycling Trials

All cycling trials were completed on a custom-built stationary cycle ergometer while participants remained in a seated position (visually checked by the experimenter). The stationary cycle ergometer was fitted with a heavy flywheel and an 11-speed hub gearing system (Alfine SG-S700, Shimano, Osaka, Japan) that was used to manipulate the resistance and therefore the cadences reached by the participants during maximal cycling efforts. Individual seat height (109% inseam height) and handlebar position were replicated across visits. The stationary cycle ergometer was fitted with 172.5 mm instrumented cranks (Axis, Swift Performance Equipment, Queensland, Australia) interfaced with clipless pedals (SPD-SL, Shimano). Crank angle, crank torque and radial force signals were recorded from each crank at 100 Hz on a computer using the manufacturer software (Swift Performance Equipment). For the maximal cycling trial, participants accelerated the flywheel as fast as possible for 10 s from a stationary start against a resistance that was controlled using the gear ratio. Strong verbal encouragement was provided to the participants.

#### Fatiguing Task

The fatiguing task was performed on an isokinetic dynamometer (Cybex Norm, NY, USA) fitted with an extension arm designed to allow bilateral contractions of the quadriceps. Participants were seated in the dynamometer chair with straps across the thorax and pelvis, while hip angle was set at 90° flexion through adjustments of the back-support orientation. The dynamometer arm was positioned ~2 cm proximal to the lateral malleoli of the fibula. The fatiguing task consisted of 120 maximal bilateral contractions of the quadriceps during concentric knee extension exercise performed in isokinetic mode (angular velocity = 15°.s^−1^; knee range of motion = 100–30° flexion with 0° = full extension). Each contraction required participants to produce a maximal effort during the concentric phase only, whilst the experimenter quickly re-set the knee angle back to 100° flexion before the start of the next contraction (work/rest ratio = 3/1). After each block of 30 contractions, the participants rested passively for 120 s. Torque (N·m), joint angle (°) and angular velocity (°.s^−1^) were continuously recorded at 100 Hz through the dynamometer software (Humac, 2009 version 10, MA, USA) during the fatiguing task. Strong verbal encouragement was provided to the participants.

#### Neuromuscular Testing (NMT)

NMT was completed while participants were seated on the chair of the isokinetic dynamometer used for the fatiguing task. A custom designed apparatus was used to measure left quadriceps isometric maximal voluntary contraction (IMVC) torque and muscle twitches evoked through supramaximal electrical stimulation of the femoral nerve. A steel threaded rod created a non-compliant connection between a force transducer (AMS-1 S Type, AWE, Ingleburn, Australia) and a padded strap fixed ~2 cm proximal to the lateral malleolus of the fibula. The orientation and length of the threaded rod was adjusted to position the knee joint at 90° flexion. A constant-current electrical stimulator (model DS7AH, Digitimer, Welwyn Garden City, UK) delivered single, supramaximal, electrical pulses (0.2 ms duration) to the femoral nerve. A hand-held dome-shaped cathode (diameter 20 mm) was pressed firmly into the proximal portion of the femoral triangle while a self-adhesive rectangular anode (90 × 50 mm) was positioned in the gluteal fold. Stimuli were delivered when the torque plateaued during the IMVC, and again 2 s after the end of the contraction when the muscle group was fully relaxed. The electrical stimuli were delivered at 1.5 × the intensity required to elicit a maximal resting twitch torque (231 ± 25 mA). The analog signal of the force transducer (amplification = 500) and trigger output from the stimulator were digitized using wireless sensors (Direct Transmission System [DTS] Universal Analog Input Probe, Noraxon Inc., AZ, USA) and recorded at 3000 Hz using MyoResearch software (Noraxon Inc., AZ, USA).

#### Electromyographic (EMG) Activity

We recorded interference EMG signals using wireless DTS EMG Probes (Noraxon Inc., AZ, USA). Disposable pre-gelled Ag-AgCl surface electrodes (Blue sensor N, Ambu, Ballerup, Denmark) were positioned 20-mm apart and aligned parallel to the underlying muscle fibers. Prior to securing the sensors and electrodes using tape, the skin was prepared by shaving all hair, lightly abrading, and cleaning with alcohol. The EMG signals were pre-amplified (×500) and digitized by the sensors before being transmitted and recorded at 3,000 Hz using MyoResearch software. During maximal cycling trials, EMG signals were recorded bilaterally from the *vastus lateralis* (VL), *vastus medialis* (VM), *rectus femoris* (RF) and *biceps femoris* (HAM), but also unilaterally (left side) from *gluteus maximus* (GMAX), *gastrocnemius medialis* (GAS), *soleus* (SOL) and *tibialis anterior* (TA). During the fatiguing task, EMG signals were recorded bilaterally from VL, VM, RF, and HAM.

### Data Analysis

All signals were processed using Visual 3D software version 5 (C-Motion Inc., Germantown, USA).

#### Maximal Cycling Trials

All mechanical signals recorded via the instrumented cranks were low-pass filtered at 10 Hz using a second-order Butterworth filter. Total pedal forces (F_tot_) applied to the left and right pedals were calculated using trigonometry. For each side and each pedal revolution of the maximal cycling trials, we extracted average power produced by each leg over the extension (top dead center to bottom dead center) and flexion phases (bottom dead center to top dead center). All power values calculated during maximal cycling in absence of fatigue were compared to the power-cadence relationship obtained during the Force-Velocity test of the corresponding participant to verify that the participants produced near-maximal levels of power with both legs through extension and flexion phases and across all pedal revolutions (Gardner et al., 2009). Forty power-cadence relationships were modeled from the data collected during the Force-Velocity test (i.e., 10 participants × 2 legs × 2 phases) using the methods previously described (Dorel et al., 2005; Rudsits et al., 2018). From the data recorded during the Force-Velocity test, we selected the gear ratio allowing each participant to perform at cadences ranging from ~15 to ~90 rpm during a 10-s maximal cycling effort performed with no fatigue, ensuring a maximal contribution from the quadriceps to total power production (McDaniel et al., 2014). Additionally, we calculated left/right power ratio over the extension phase and the flexion phase to verify that the effect of the fatiguing task was evenly distributed across the two legs.

All raw EMG signals recorded during maximal cycling trials were band-pass filtered (20–500 Hz), full wave rectified, and smoothed using a 7 Hz low-pass fourth order Butterworth filter to create linear envelopes (O'Bryan et al., 2014). The amplitude of these EMG signals was normalized to the peak EMG recorded during the maximal cycling effort performed at the end of the cycling warm-up during the same visit (Rouffet and Hautier, 2008; O'Bryan et al., 2014). An average EMG profile was calculated for VL and VM (VAS), while EMG profiles of the other muscles were considered separately (O'Bryan et al., 2014, 2017). Finally, we assessed variability in total force (F_tot_) applied to the two pedals and EMG activity of the muscles across the participants by calculating variance ratios (VR) using the following equation:


$$\begin{array}{l} \;VR=\;\frac{\sum_{i=1}^{k}\sum_{j=1}^{n}{\left(X_{ij}-{\bar{X}}_{i}\right)}^{2}/k\left(n-1\right)}{{\sum_{i=1}^{k}\sum_{j=1}^{n}\left(X_{ij}-\bar{X}\right)}^{2}/\left(kn-1\right)}\\ Where;\bar{X}=\frac{1}{k}\sum_{i=1}^{k}{\bar{X}}_{i} \end{array}$$

Where, *k* is equal to the number of intervals considered over the pedal cycle (*k* = 101); *n* is equal to the total number of subjects (*n* = 10); *X*_*ij*_ corresponds to the value at the *ith* interval for the *jth* subject; and ${\bar{X}}_{i}$ is the average value for the *ith* interval calculated over the *n* subjects (Rouffet et al., 2009). VR values were calculated for each pedal and muscle during the maximal cycling efforts performed in both conditions (no fatigue and post-fatiguing task).

#### Fatiguing Task and Neuromuscular Testing (NMT)

From the torque signal recorded from the isokinetic dynamometer during the fatiguing task, we calculated an average of the peak torque values produced by the participants during the three initial and the three final contractions. During NMT, IMVC torque of the left quadriceps was calculated as an average of the torque measured over a 500-ms window immediately preceding the supramaximal electrical stimulus delivered to obtain the superimposed twitch (SIT) (Babault et al., 2006). Peak potentiated resting twitch torque (RT) and peak-to-peak amplitude of the compound muscle action potential (M-wave) for VL and VM were calculated from the electrically-evoked response elicited 2 s after IMVC while the quadriceps muscles were relaxed. Voluntary activation (VA) was calculated using the equation: VA = (1 – SIT/RT) × 100 as previously established (Taylor et al., 2009; D'Amico et al., 2020).

### Statistics

Statistical analysis was completed using SPSS software (version 22, Chicago, IL, USA). Normal distribution of the data was examined via a Shapiro-Wilk test, with a transformation applied to data violating this assumption depending on the skew. Repeated measures one-way ANOVA compared left quadriceps IMVC torque before, 5 s and 60 s after the end of the fatiguing task (visit #3), and NMT variables (i.e. IMVC torque, VA, SIT, and RT) before and 5 s after the fatiguing task (visit #4 or #5). A series of two-way ANOVAs with repeated measures were used to test; (1) the effect of condition and side on the mechanical and EMG variables recorded bilaterally during maximal cycling, (2) the effect of condition and phase on mechanical variables during maximal cycling, and (3) the effect of condition and muscles on the peak EMG during maximal cycling. A non-parametric Wilcoxon Signed Rank Test examined the effect of condition on inter-individual variance ratios calculated for EMG and force profiles, as the assumption of normality was still violated after data transformation. A series of Pearson product-moment correlations were used to examine the associations between left quadriceps IMVC torque and average power produced over the extension and flexion phases of the left leg during maximal cycling without and with quadriceps fatigue, and between reductions in VA and RT with the decreases in knee extension IMVC torque after the fatiguing task. Effect sizes were calculated using partial eta squared (η^2^P) for repeated measures ANOVAs (small = 0.01 - 0.059, moderate = 0.06–0.139, large ≥ 0.14) and Cohen's *d* for paired comparisons (*d*: trivial = 0–0.19, small: 0.2–0.49, moderate: 0.5–0.79, large: > 0.8). All *post-hoc* and paired comparisons were Bonferroni adjusted. The significance level was set to *P* < 0.05 and results are reported as means ± SD, unless indicated otherwise.

## Results

### Neuromuscular Fatigue of the Quadriceps

Bilateral quadriceps torque values at the start and end of the fatiguing task were similar during visit #3 and visit #4 or #5 (*F*_1,27_ = 1.303; *P* = 0.264; η^2^P = 0.05). Quadriceps IMVC measured from NMT during visit #3 showed an effect of time (*F*_2,18_ = 26.759; *P* < 0.001; η^2^P = 0.75), with higher quadriceps IMVC torque recorded before the fatiguing task (169 ± 57 N·m) compared to 5 s (70 ± 29 N·m, *d* = 1.47) and 60 s (77 ± 29 N·m, *d* = 1.43) after completion of the fatiguing task. Importantly, no significant difference in quadriceps IMVC torque between 5 and 60 s after the end of the fatiguing task was identified (*P* = 0.698).

**Figure 2:**
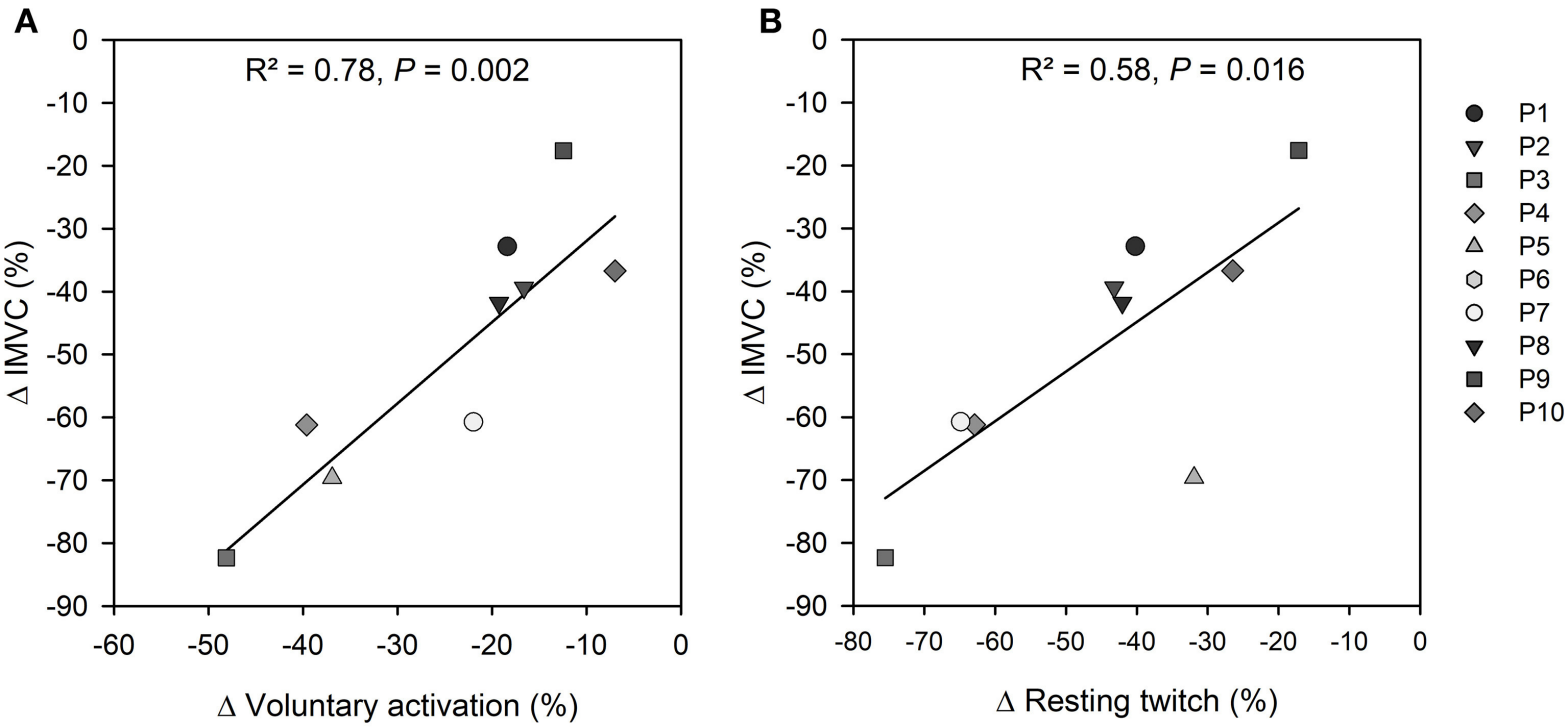
Completion of the fatiguing task led to a reduction in quadriceps IMVC (ranging from −18 to −82%) that was associated with **(A)** a decrease in voluntary activation (ranging from −7 to −48%); and **(B)** a decline in resting twitch (ranging from −17 to −76%). *P#6 was excluded because of a technical issue with VA measurement after the fatiguing task*.

From the data collected during NMT performed before and 5 s after the fatiguing task preceding the maximal cycling trial, quadriceps IMVC torque was reduced (165 ± 60 vs. 78 ± 33 N·m; *P* = 0.002, *d* = 1.34), RT size decreased (48 ± 15 vs. 25 ± 8 N·m; *P* = 0.002, *d* = 1.37), SIT size increased (4.4 ± 2.3 vs. 8.4 ± 2.6 N·m; *P* = 0.002, *d* = −1.27), voluntary activation was reduced (91 ± 4 vs. 67 ± 14%; *P* < 0.001, *d* = 1.52), and M-wave amplitude was unchanged for VL (8.7 ± 3.8 mV vs. 8.9 ± 4.1 mV; *P* = 0.58, *d* = −0.07) and VM (10.4 ± 2.4 mV vs. 11.0 ± 3.0 mV; *P* = 0.15, *d* = −0.2). Results from linear regression analyses showed that the decrease in quadriceps IMVC torque was associated with reductions in both VA (R^2^ = 0.78, *P* = 0.002) and RT (R^2^ = 0.58, *P* = 0.016) (Figures 2A,B).

### Peak EMG Activity During Maximal Cycling

The peak EMG values during maximal cycling in absence of fatigue ranged from ~80 to ~95% of the values recorded during maximal cycling at the end of the warm-up. When considering the eight muscles recorded from the left leg, we observed a muscle effect (*F*_7,49_ = 4.5; *P* < 0.001; η^2^P = 0.39) and a condition effect (*F*_1,7_ = 13.8; *P* < 0.01; η^2^P = 0.66), but no muscle × condition interaction (*F*_7,49_ = 1.3; *P* = 0.28; η^2^P = 0.15), with peak EMG decreasing by ~10% across the different muscles (Table 1). When considering muscles recorded bilaterally, peak EMG values were similarly lowered in VAS (*F*_1,19_ = 33.1, *P* < 0.001; η^2^P = 0.64), RF (*F*_1,8_ = 10.6, *P* < 0.05; η^2^P = 0.57) and HAM muscles (*F*_1,7_ = 21.7, *P* < 0.01; η^2^P = 0.76) following the fatiguing task.

**Table 1:** Peak EMGs were reduced across all lower limb muscles during maximal cycling with fatigued quadriceps muscle.

	**Left side**					**Right side**				
	**No fatigue**	**Fatigued quadriceps**	**Muscle effect**	**Condition effect**	**Muscle × condition**	**No fatigue**	**Fatigued quadriceps**	**Side effect**	**Condition effect**	**Side × condition**
VAS	90 ± 7	78 ± 10	***P*** **<** **0.001**	***P*** **<** **0.01**	*P* = 0.28	89 ± 7	76 ± 9	*P* = 0.3	***P*** **<** **0.001**	*P* = 0.68
RF	93 ± 3	83 ± 13				92 ± 8	77 ± 19	*P* = 0.28	***P*** **<** **0.05**	*P* = 0.06
HAM	83 ± 9	67 ± 12				84 ± 11	72 ± 15	*P* =0.16	***P*** **<** **0.01**	*P* = 0.41
GMAX	87 ± 6	76 ± 16								
GAS	85 ± 9	83 ± 16								
SOL	93 ± 7	84 ± 11								
TA	87 ± 8	72 ± 13								

*Results are reported as mean ± SD. Significant effects values were highlighted in bold*.

### Power Production During Maximal Cycling

During maximal cycling in the absence of fatigue, participants produced near maximal levels of power using both legs and over the extension and flexion phases (~95 and ~90% of cadence specific maximal power, respectively) of the pedaling movement.

**Figure 3:**
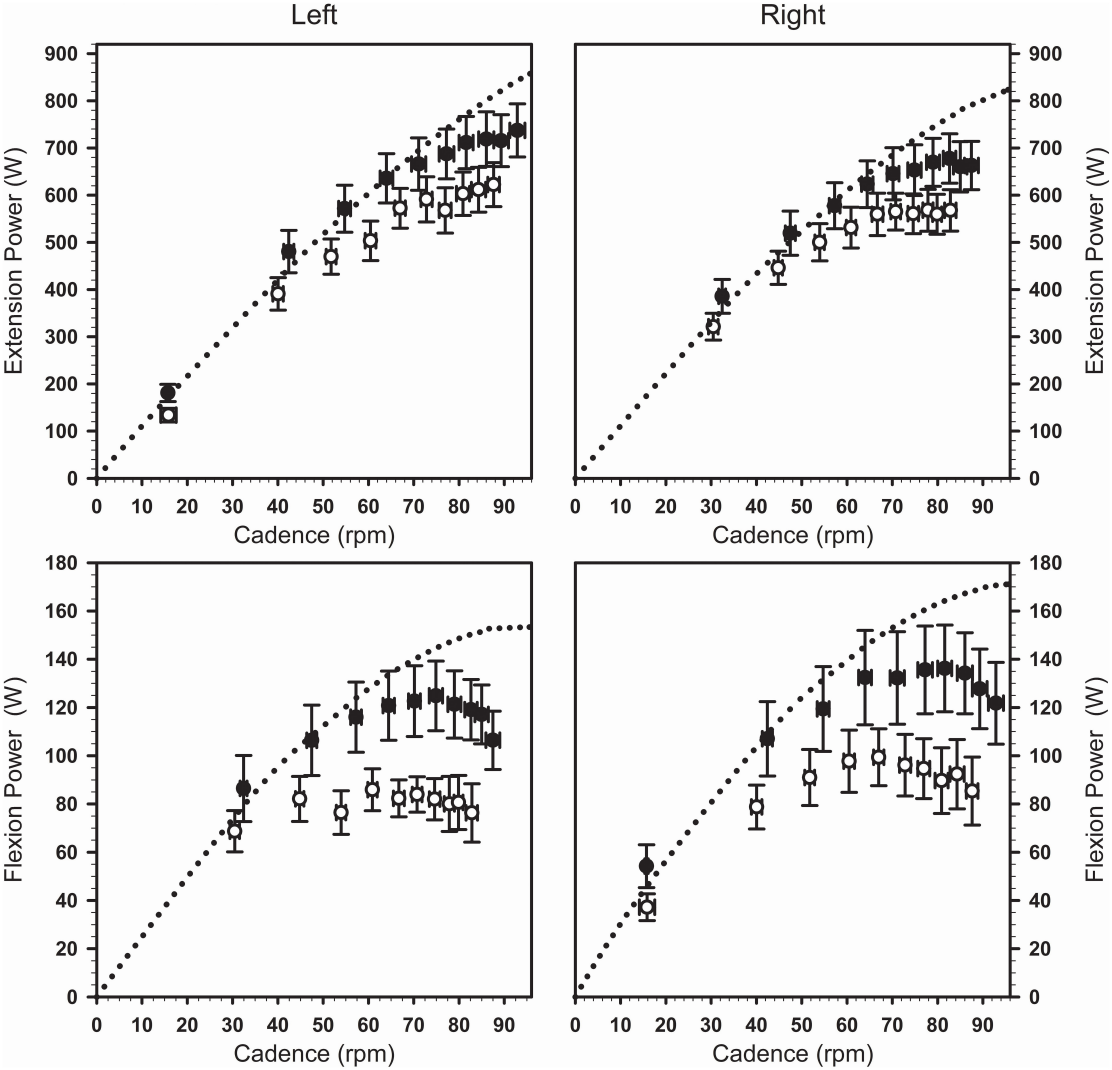
Changes in cadence and power (average ± SEM) seen across the maximal cycling trials completed in the absence of fatigue (black symbols) and following the fatiguing task (white symbols). The dotted curves show the average power-cadence relationships obtained from the Force-Velocity test for each leg and each phase of the pedaling movement. Both maximal cycling trials were initiated with the left crank positioned just before top dead center while the same external resistance was used across both conditions.

During maximal cycling after the fatiguing task, power was produced at lower cadences (*F*_1,19_ = 45.3; *P* < 0.001; η^2^P = 0.71; Figure 3). Absolute power production was reduced over both the extension phase (*F*_1,9_ = 14.8; *P* < 0.01; η^2^P = 0.62) and the flexion phase (*F*_1,9_ = 15.2; *P* < 0.01; η^2^P = 0.63), with comparable changes across the legs as indicated by a lack of condition × side interaction (Table 2). A condition × phase interaction revealed greater relative reductions in power for the flexion phase compared to the extension phase (−24 ± 18 vs. −15 ± 8%, *F*_1,19_ = 21.4; *P* < 0.001; η^2^P = 0.53). Similar left/right power ratios during the extension and the flexion phases were calculated for the maximal cycling trials performed in the absence of fatigue and after the fatiguing task (*F*_1,9_ = 1.0; *P* = 0.34; η^2^P = 0.10).

**Table 2:** Participants produced near maximal levels of power during maximal cycling in absence of fatigue over the extension and flexion phases of the pedal cycle.

	**Left side**		**Right side**		
	**No fatigue**	**Fatigued quadriceps**	**No fatigue**	**Fatigued quadriceps**	***P* values**
Extension power (W)	635 ± 164	525 ± 128	607 ± 156	516 ± 122	Side: 0.70 **Cond:** **<** **0.01** Int: 0.82
Flexion power (W)	114 ± 41	82 ± 26	120 ± 52	86 ± 33	Side: 0.40 **Cond:** **<** **0.01** Int: 0.56
Extension power (%)	95 ± 5	84 ± 7	94 ± 4	84 ± 7	Side: 0.45 **Cond:** **<** **0.001** Int: 0.22
Flexion Power (%)	88 ± 9	68 ± 21	87 ± 6	69 ± 16	Side: 0.87 **Cond:** **<** **0.01** Int: 0.63

*Following the fatiguing task, we observed reductions extension power and flexion power across both legs. Significant effects values were highlighted in bold*.

### Relationships Between Quadriceps IMVC Torque and Power Production During Maximal Cycling

**Figure 4:**
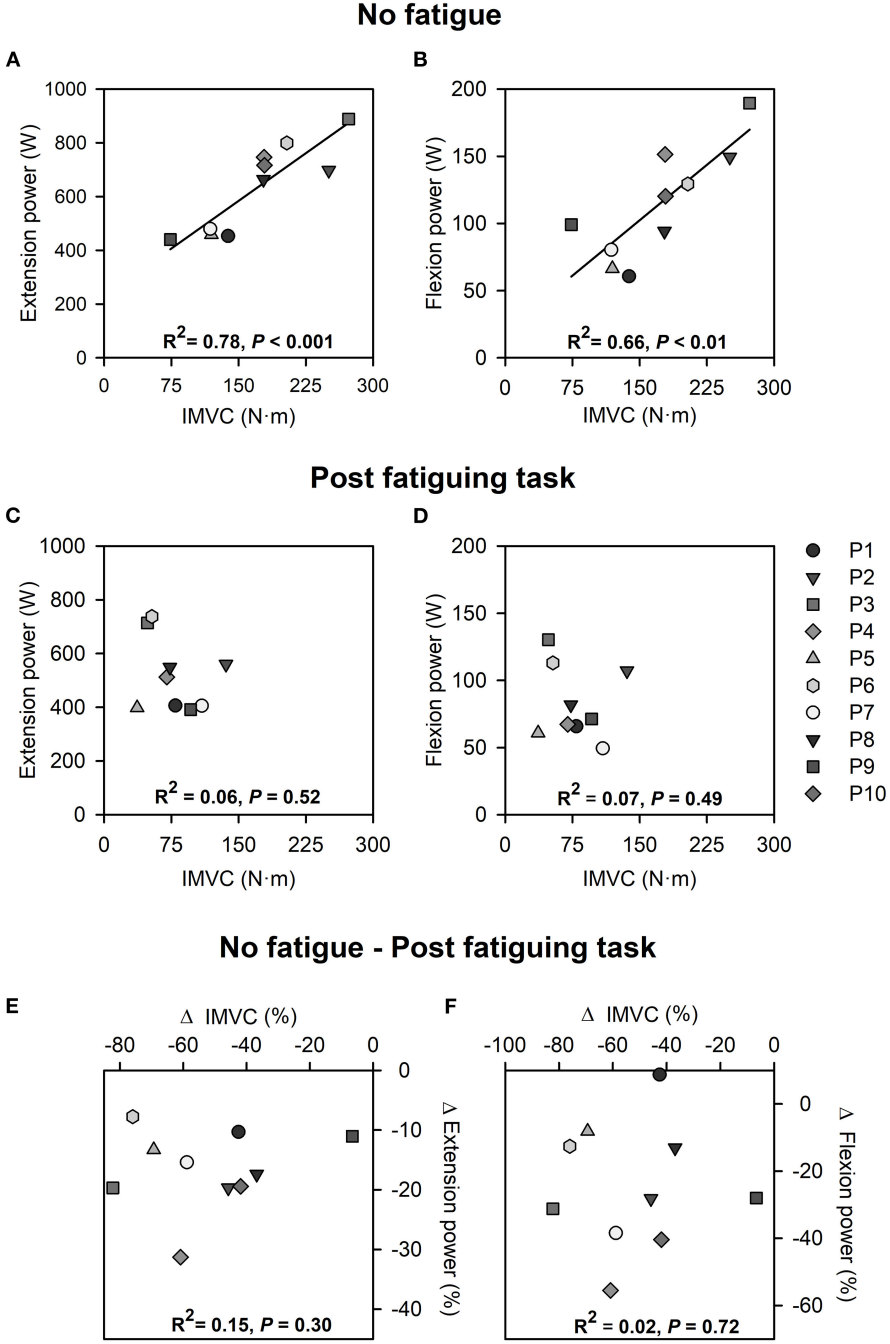
Correlations between quadriceps IMVC and extension power **(A)** and flexion power **(B)** during maximal cycling in the absence of quadriceps fatigue. Correlations between quadriceps IMVC and extension power **(C)** and flexion power **(D)** during maximal cycling with quadriceps fatigue. Correlations between changes in quadriceps IMVC and changes in extension power **(E)** and flexion power **(F)** during maximal cycling following the fatiguing task. All analyses were performed from data collected from the left side.

In the absence of fatigue, maximal quadriceps IMVC torque was correlated with average power produced over the extension phase (*R*^2^ = 0.78; *P* < 0.001) and average power produced over the flexion phase (*R*^2^ = 0.66; *P* < 0.01) during the subsequent maximal cycling trial (Figures 4A,B). After the fatiguing task, no associations between maximal quadriceps IMVC torque and power produced over the extension phase (*R*^2^ = 0.06; *P* = 0.52), nor power produced over the flexion phase (*R*^2^ = 0.07; *P* = 0.49) were observed (Figures 4C,D). Moreover, the relative reductions in quadriceps IMVC torque were not associated with relative reductions in average power over the extension phase (*R*^2^ = 0.15; *P* = 0.30), nor average power over the flexion phase (*R*^2^ = 0.02; *P* = 0.72) (Figures 4E,F).

### Variability of Pedal Force and EMG Profiles During Maximal Cycling

**Figure 5:**
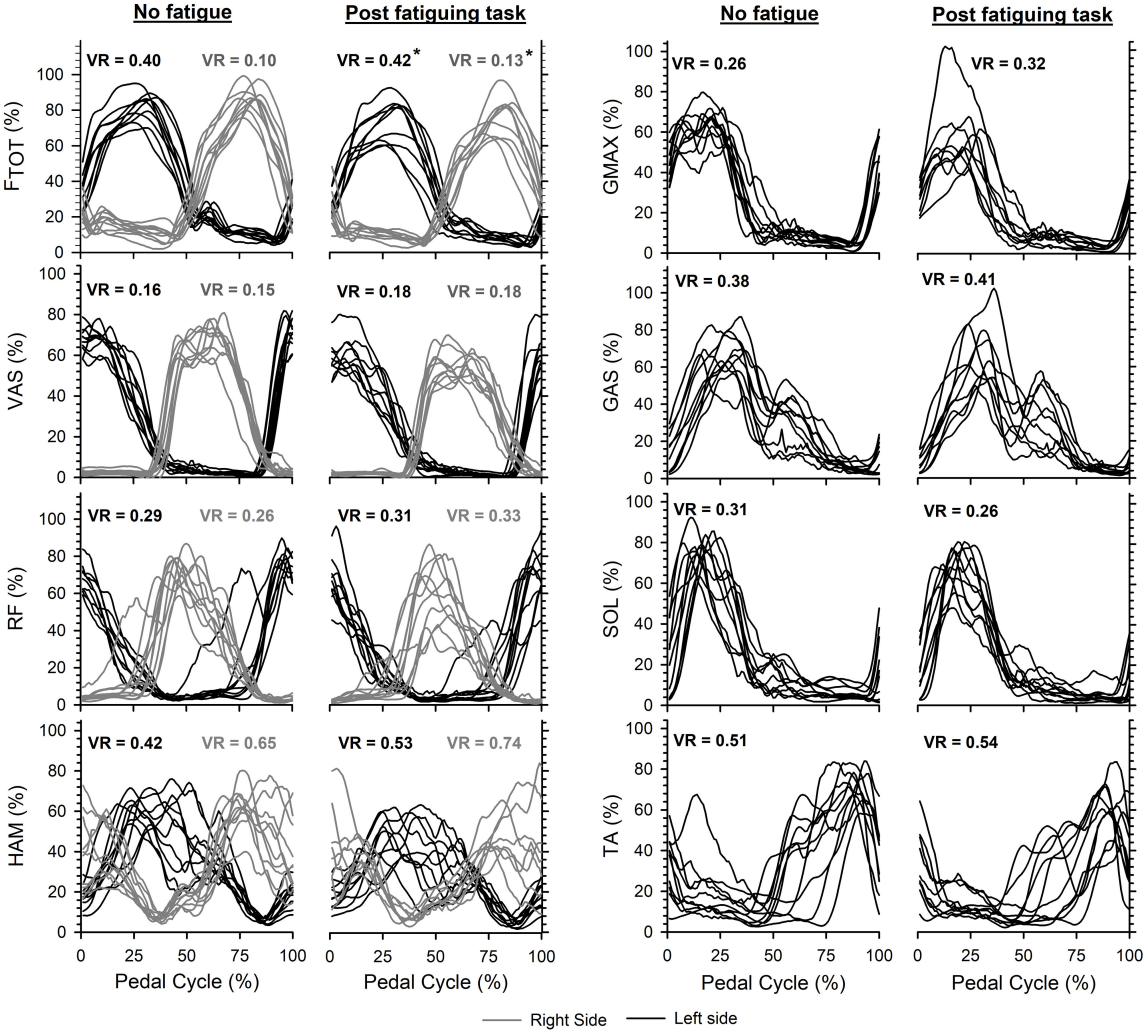
Average profiles for total pedal force (F_tot_) and EMG activity obtained for each participant on the left side (black curves) and right side (gray curves) during maximal cycling without and with quadriceps fatigue. Note that EMG for seven muscles was recorded on the left, and only three muscles on the right. Larger variance ratios (VR) were calculated for F_tot_ (* indicates *P* < 0.05) and across the different EMG profiles (*P* < 0.05) after the fatiguing task. The start/end of the pedal cycles were defined when the left crank was at top dead center while maximal cycling trials were initiated with the left crank positioned just before top dead center.

Increased variance ratios were calculated for the EMG profiles of the eight lower limb muscles collectively (*P* < 0.001) and the F_tot_ profiles (Left: *P* = 0.028 and Right: *P* = 0.005) during maximal cycling after the fatiguing task (Figure 5).

## Discussion

The first aim of this study was to determine the effect of a series of bilateral and voluntary knee extensions on quadriceps fatigue as well as activation of the lower-limb muscles and power production during a maximal cycling effort. The voluntary contractions caused both a reduction of voluntary activation and impairments of the contractile properties of the quadriceps that resulted in a ~50% group average decrease in the quadriceps IMVC. Quadriceps fatigue was accompanied by ~10% reduction in peak EMG across all lower-limb muscles during subsequent maximal cycling. Altogether, these changes resulted in a decrease of power production over the extension (~15%) and the flexion (~24%) phases. The second aim was to investigate the association between quadriceps IMVC and power production over the extension and flexion phases during maximal cycling with fatigued quadriceps. Knee extensors IMVC and power production during maximal cycling were well correlated prior to fatigue but no longer correlated after the fatiguing task. Furthermore, there was no association between the reductions in power production during maximal cycling and the reductions in quadriceps IMVC.

### Quadriceps Fatigue After the Voluntary Contractions

Completion of 120 voluntary contractions led to large levels of fatigue in both quadriceps, as shown by similar reductions in knee extension torque measured at the end of the fatiguing task and 5-s later during NMT. As anticipated, quadriceps muscle fatigue resulted from central and peripheral mechanisms. The central component of fatigue was evidenced by a decrease in VA (Figure 2A), reflecting a reduced ability of the participants to voluntarily activate their quadriceps muscles (Morel et al., 2015). The peripheral component of fatigue was evidenced by a reduction in the amplitude of the resting twitch (Figure 2B), whereas no major changes in sarcolemma excitability of VL and VM were identified from analysis of M-wave amplitudes. As quadriceps IMVC torque, VA and RT values were similar when measured 5 and 60 s after the end of the fatiguing task during Visit #3 (see also Cheng and Rice, 2005; Froyd et al., 2013), the changes in power production and muscle activation during maximal cycling after the end of the fatiguing task were interpreted in light of reduced voluntary activation and impairments of the contractile properties of the quadriceps muscles. A limitation of our study is that changes in voluntary activation and the resting twitch represent the sum of responses from all the knee extensors so that we could not dissociate the etiology and magnitude of fatigue in the VAS and RF muscles.

### Changes in EMG Activity During Maximal Cycling

As shown in Table 1, VAS and RF muscles were near-maximally activated during maximal cycling in the absence of fatigue (Rouffet and Hautier, 2008; Dorel et al., 2012). The reduced EMG activity seen in VAS and RF muscles during maximal cycling with fatigued quadriceps seems related to the reduction in voluntary activation of this muscle group after the fatiguing task, as we did not detect changes in M waves. Because the fatiguing task consisted of voluntary contractions, VA reductions are likely to be associated with reduced excitability of the motoneurones innervating the quadriceps (Finn et al., 2018; D'Amico et al., 2020) and inadequate output from the motor cortex (Goodall et al., 2010; Takahashi et al., 2011; Sidhu et al., 2014). In contrast with previous findings reported following quadriceps fatigue induced using electrostimulation (Brochner Nielsen et al., 2018), we observed a reduction of peak EMG across all the lower-limb muscles during maximal cycling after a series of fatiguing voluntary contractions. This discrepancy is likely due to the development of central fatigue that resulted from the voluntary contractions but not from the electrostimulation protocol used by Brochner Nielsen et al. (2018). Additionally, the reduced activation of all lower limb muscles could reflect an adjustment made by the supraspinal centers to maintain whole-body homeostasis (Lambert et al., 2005) when maximal cycling is performed with high levels of peripheral fatigue in a large muscle group. Finally, the absence of substantial changes in the EMG patterns aside from the reductions in peak EMG may be interpreted as evidence that the spinal central pattern generators retained control over the coordination pattern (Raasch and Zajac, 1999; Hug et al., 2010).

### Changes in Extension Power During Maximal Cycling

In line with previous findings (Driss et al., 2002; Kordi et al., 2017), knee extension IMVC was a predictor of power production over the extension phase during maximal cycling in absence of fatigue, (Figure 4A). After the fatiguing task, we observed a ~15% group average decrease in extension power during maximal cycling (Figure 3), which likely resulted from the impact of reduced voluntary activation and impairments of the contractile properties of the quadriceps muscles (Figure 2). The amount of energy directly transmitted by VAS muscles to the cranks over the extension phase was likely reduced following the fatiguing task (van Ingen Schenau, 1989; Raasch et al., 1997; McDaniel et al., 2014). Importantly, the decreased activation of GMAX, HAM, GAS and SOL muscles (Table 1) also likely contributed to reducing the energy transferred from the limb to the pedal over the different portions of the extension phase.

### Changes in Flexion Power During Maximal Cycling

During maximal cycling after the fatiguing task, we observed a ~24% group average decrease in flexion power (Figure 3) which can also be partly explained by reduced voluntary activation and impairments of the contractile properties of RF muscle (Figure 2). Because the bi-articular muscle presents a higher percentage of type II muscle fibers (~62 vs. ~54%) compared to the VAS muscles (Johnson et al., 1973), it is possible that RF muscle experienced more severe impairments of the its contractile properties compared to VAS so that the force-generating capacity of the bi-articular muscle was further reduced. In addition to central and peripheral fatigue of RF muscle, we observed a decreased activation of HAM and TA muscles (Table 1). Our results are in agreement with the decreased EMG activity reported in the hamstring muscles that was associated with fatigue-related increases of firing of group III/IV afferents in the ipsilateral knee extensors (Amann et al., 2011; Kennedy et al., 2015). Central and peripheral fatigue of RF combined with a reduced activation of HAM and TA likely reduced energy transferred from the limb to the pedal over the flexion phase (Raasch et al., 1997). Interestingly, we observed a larger decrease in flexion power compared to extension power when expressed in relative terms (~24 vs. ~15%; see Table 2). To explain the larger relative reduction in flexion power, we propose that RF muscle experienced larger levels of peripheral fatigue than VAS muscles and that RF makes a larger contribution to the mechanical energy transmitted to the crank over the flexion phase compared to the contribution made by VAS over the extension phase. Another limitation of our study is that we did not record the kinematics of the lower limb segments, preventing us from the opportunity to study the effect of fatigue on the various joint powers (Martin and Brown, 2009).

### Association Between Quadriceps IMVC and Power During Maximal Cycling

In line with previous reports, we observed positive associations between quadriceps IMVC and extension power but also flexion power produced during maximal cycling in absence of fatigue (see Figures 4A,B, respectively). To the best of our knowledge, the association between quadriceps IMVC and flexion power has not been previously reported and may be explained by the large RF muscle contribution (~50%) to the energy directly transmitted to the cranks over the flexion phase during maximal cycling in the absence of fatigue (van Ingen Schenau, 1989; Raasch et al., 1997; McDaniel et al., 2014). However, quadriceps fatigue abolished these associations so that the individuals able to produce larger quadriceps forces following the fatiguing task were not necessarily those who could produce higher levels of power during either the extension or the flexion phases of maximal cycling (see Figures 4C,D). Additionally, the magnitude of the changes in quadriceps IMVC was not associated with the magnitude of the changes in power produced over the extension and flexion phases during maximal cycling after the fatiguing task (Figures 4E,F). These results may be explained by increased inter-individual variability of the force and EMG profiles during maximal cycling with fatigued quadriceps (Figure 5). Each participant may have adjusted the activation of their lower limb muscles differently according to the motor abundance offered by their musculoskeletal system (Latash, 2012) and the individuals' prior experience with similar exercises (Ting et al., 2015). For example, some individuals may have tried to maximize extension power through activation of the gluteus maximus, while others attempted to maximize flexion power at the expense of extension power. The dissociation between quadriceps IMVC and power production during maximal cycling could also be explained by discrepancies across individuals in terms of the distribution of the power generating capacities among the lower-limb muscles. For example, we can expect differences in the ratio between the force generating capacity of the GMAX and VAS across individuals, which would likely affect the decrease in extension power caused by quadriceps fatigue (Martin and Brown, 2009).

To conclude, we observed that pronounced levels of fatigue in the quadriceps resulting from central and peripheral mechanisms of fatigue reduced activation of all lower-limb muscles and decreased power production by ~15% during the extension phase and ~24% during the flexion phase during maximal cycling. We postulate that larger relative reduction in flexion power during maximal cycling with fatigued quadriceps muscles can be explained by larger impairments of the contractile properties for RF associated with a higher contribution of quadriceps muscles to the mechanical energy transmitted to the crank over the flexion phase compared to the extension phase. Quadriceps fatigue also led to increased inter-individual variability of the total force and the EMG profiles of most lower-limb muscles, which likely also contributed to reducing power production during maximal cycling (Rudsits et al., 2018). Ultimately, the reductions in extension and flexion power during maximal cycling were not correlated with the reductions in quadriceps IMVC measured through knee extension, providing another illustration of how motor redundancy affects performances produced during complex movements.

## Data Availability Statement

The raw data supporting the conclusions of this article will be made available by the authors, upon request.

## Ethics Statement

The studies involving human participants were reviewed and approved by Victoria University Human Research Ethics Committee (VUHREC). The patients/participants provided their written informed consent to participate in this study.

## Author Contributions

DR, JT, JD'A, and SO'B contributed to conception and design of the study. SO'B collected the data and performed the statistical analysis. SO'B and DR wrote the first draft of the manuscript. SO'B, JT, and DR wrote sections of the manuscript. All authors contributed to manuscript revision, read, and approved the submitted version.

## Funding

Funding was received for SO'B through an Australian Postgraduate Award. Funding was received from the Institute for Health and Sport (IHeS) at Victoria University for the open access publication fees.

## Conflict of Interest

The authors declare that the research was conducted in the absence of any commercial or financial relationships that could be construed as a potential conflict of interest.

## Publisher's Note

All claims expressed in this article are solely those of the authors and do not necessarily represent those of their affiliated organizations, or those of the publisher, the editors and the reviewers. Any product that may be evaluated in this article, or claim that may be made by its manufacturer, is not guaranteed or endorsed by the publisher.
